# 

**Published:** 1886-01

**Authors:** 


### CONTENTS:

Art. I—Diseases of the Period of Dentition. By W. C. Barrett, M. D., D. D. S........385

Art. II—First District Dental Society, State of New York........................406

Art. III—Hydrochlorate of Cocaine. By Dr. Geo. Watt..........................422

### EDITORIAL, ETC.

Forty Years Ago.—Mode of Treating Pulpless and Abscessed Teeth, after the Pulp has been suppurated, or where it has been destroyed with a view to this Operation. By Samuel Rambo, M. D., of Montgomery, Ala........................................425

The International Medical Congress ..........................................428

Oral and Dental Section of the Ninth International Medical Congress................429

### MONTHLY SUMMARY.

Disgust for Decayed Teeth.................................................431
Zinc Chloride as a Disinfectant.......................... ......................432

